# Stress Response of *Vibrio parahaemolyticus* and *Listeria monocytogenes* Biofilms to Different Modified Atmospheres

**DOI:** 10.3389/fmicb.2020.00023

**Published:** 2020-02-20

**Authors:** Hui Qian, Wei Li, Linxia Guo, Ling Tan, Haiquan Liu, Jingjing Wang, Yingjie Pan, Yong Zhao

**Affiliations:** ^1^College of Food Science and Technology, Shanghai Ocean University, Shanghai, China; ^2^Laboratory of Quality and Safety Risk Assessment for Aquatic Products on Storage and Preservation (Shanghai), Ministry of Agriculture, Shanghai, China; ^3^Shanghai Engineering Research Center of Aquatic-Product Processing and Preservation, Shanghai, China; ^4^Engineering Research Center of Food Thermal-Processing Technology, Shanghai Ocean University, Shanghai, China

**Keywords:** stress response, *Vibrio parahaemolyticus*, *Listeria monocytogenes*, modified atmospheres, biofilms, extracellular polymeric substance, gene expression

## Abstract

The sessile biofilms of *Vibrio parahaemolyticus* and *Listeria monocytogenes* have increasingly become a critical threat in seafood safety. This study aimed to evaluate the effects of modified atmospheres on the formation ability of *V. parahaemolyticus* and *L. monocytogenes* biofilms. The stress responses of bacterial biofilm formation to modified atmospheres including anaerobiosis (20% carbon dioxide, 80% nitrogen), micro-aerobiosis (20% oxygen, 80% nitrogen), and aerobiosis (60% oxygen, 40% nitrogen) were illuminated by determining the live cells, chemical composition analysis, textural parameter changes, expression of regulatory genes, etc. Results showed that the biofilm formation ability of *V. parahaemolyticus* was efficiently decreased, supported by the fact that the modified atmospheres significantly reduced the key chemical composition [extracellular DNA (eDNA) and extracellular proteins] of the extracellular polymeric substance (EPS) and negatively altered the textural parameters (biovolume, thickness, and bio-roughness) of biofilms during the physiological conversion from anaerobiosis to aerobiosis, while the modified atmosphere treatment increased the key chemical composition of EPS and the textural parameters of *L. monocytogenes* biofilms from anaerobiosis to aerobiosis. Meanwhile, the expression of biofilm formation genes (*luxS*, *aphA*, *mshA*, *oxyR*, and *opaR*), EPS production genes (*cpsA*, *cpsC*, and *cpsR*), and virulence genes (*vopS*, *vopD1*, *vcrD1*, *vopP2*β, and *vcrD2*β) of *V. parahaemolyticus* was downregulated. For the *L. monocytogenes* cells, the expression of biofilm formation genes (*flgA*, *flgU*, and *degU*), EPS production genes (*Imo2554*, *Imo2504*, *inlA*, *rmlB*), and virulence genes (*vopS*, *vopD1*, *vcrD1*, *vopP2*β, and *vcrD2*β) was upregulated during the physiological conversion. All these results indicated that the modified atmospheres possessed significantly different regulation on the biofilm formation of Gram-negative *V. parahaemolyticus* and Gram-positive *L. monocytogenes*, which will provide a novel insight to unlock the efficient control of Gram-negative and Gram-positive bacteria in modified-atmosphere packaged food.

## Introduction

*Vibrio parahaemolyticus* and *Listeria monocytogenes* are important facultative and food-borne pathogens in the food industry (Xu et al., 2015; Vazquez-Boland et al., 2017; Chimalapati et al., 2018). In 2014, 605 cases of *V. parahaemolyticus* infections in the United States were reported according to the Cholera and Other Vibrio Illness Surveillance (Centers for Disease Control and Prevention [CDC], 2014). Meanwhile, 675 listeriosis cases were reported in 47 states and the District of Columbia (Centers for Disease Control and Prevention [CDC], 2014). In China, 71 outbreaks of *V. parahaemolyticus* in Zhejiang Province were monitored from 2010 to 2014, resulting in 933 illnesses and 117 hospitalizations (Chen et al., 2017). For listeriosis, 253 invasive cases are reported from 2011 to 2016 in 19 provinces (Li W. et al., 2018).

In natural environments, the formation of bacterial biofilms is the prevailing microbial lifestyle (Watnick and Kolter, 2000). Biofilms are complex communities of microorganisms attached to surfaces and enclosed in firm three-dimensional, multicellular, complex, and self-assembled extracellular polymeric substances (EPS) [exopolysaccharides, proteins, extracellular DNA (eDNA), etc.] (Lamas et al., 2016; Han et al., 2017). EPS immobilizes biofilm cells, which facilitates their intense interactions including intercellular communication, horizontal gene transfer, and the formation of synergistic microconsortia (Flemming and Wingender, 2010). In addition, EPS contributes to the initial attachment and nutrient capture of bacteria and the integrity of the biofilm structures (Han et al., 2017; Tan et al., 2018). Meanwhile, EPS has been demonstrated to have good resistance to different environmental stresses including disinfectants, desiccation, salinity, temperature, heavy metal pollution, etc. (Xue et al., 2012; Blanco et al., 2019).

During recent decades, there is increasing evidence indicating that biofilms are involved in contaminating food processing equipment and food products (Brooks and Flint, 2008), including *V. parahaemolyticus* and *L. monocytogenes*. For *V. parahaemolyticus*, it can form biofilms on various biotic or abiotic surfaces such as oysters, stainless steel (SS), etc. (Mizan et al., 2016). Moreover, the pathogenic strains of *V. parahaemolyticus*, on average, formed more biofilm than non-pathogenic strains at all tested temperatures (Song et al., 2016). Additionally, *V. parahaemolyticus* formed the highest amount of biofilms at 2% NaCl and the least biofilm at 5% NaCl (Mizan et al., 2018). For *L. monocytogenes*, when bacteriocin from *L. plantarum* ST8SH, vancomycin (antimicrobial), propolis (a natural antimicrobial product), and EDTA (chelating agent) are used individually or combined, the formation of bacterial biofilms will be inhibited in different degrees (Todorov et al., 2018). Different strains differ in their biofilm formation ability, which is closely linked with the resistance of *L. monocytogenes* to antimicrobials in food processing environments (Pan et al., 2009; van der Veen and Abee, 2010). The study suggests that EDTA influences biofilm formation by affecting the initial adherence of *L. monocytogenes* onto abiotic surfaces (Chang et al., 2012).

For *Vibrio*, extracellular nucleases (Xds and Dns) control the level of eDNA and are involved in multiple processes including the development of a typical three-dimensional biofilm structure (Seper et al., 2011). The exogenous addition of extracellular flagellin-homologous proteins (rFHPs) significantly increased the biofilm formation of *V. parahaemolyticus*, reaching ∼3.8-fold compared to control (Jung et al., 2019). For timely expression of EPS under specific conditions, bacterial cells utilize diverse signal recognition systems and subsequent regulatory mechanisms, such as quorum sensing and cyclic di-GMP (c-di-GMP) (Jung et al., 2019). CpsQ is a c-di-GMP-binding transcription factor that activates the expression of capsular polysaccharide (CPS) genes, thereby inducing biofilm development in *V. parahaemolyticus* (Zhou et al., 2013). For *L. monocytogenes*, all genes of the *pssA-E* operon and a separately located *pssZ* gene are required for exopolysaccharide production. Under environmental conditions, eDNA of *L. monocytogenes* is released by lysate bacteria, which enhances the initial attachment and formation of biofilm (Colagiorgi et al., 2016). Meanwhile, the bacterial surface or extracellular proteins have been shown to be responsible for the induction of biofilm formation (Kayal and Charbit, 2006; Franciosa et al., 2009; Colagiorgi et al., 2016).

The expression of genes related to bacterial biofilm plays a key role in biofilm formation. Flagella and other filamentous structures regulated by related genes have clearly been shown to contribute to the initial approach, attachment, and efficient biofilm formation on surfaces (Kirov, 2003). The bacterial gene expression regulated by quorum sensing is caused by small molecules called autoinducers, allowing bacteria to adapt efficiently to environmental conditions during growth (Lamas et al., 2016). For *V. parahaemolyticus*, the quorum-sensing genes including *opaR* and *luxS* play a critical role in the biofilm formation. Additionally, the *cpsA* and *cpsC* gene-regulated secretion of CPS is associated with the adhesion ability of *V. parahaemolyticus* (Hsieh et al., 2003).

The stress response (such as oxygen starvation stress, etc.) is an adaptive strategy for bacteria, which allows them to rapidly cope with changing environmental conditions and ensure their survival (Schimel et al., 2007; Alvarez-Ordonez et al., 2015). When the modified atmospheres act on bacteria, they are able to maintain viability by regulating certain genes (Alvarez-Ordonez et al., 2015). Previous studies found that *V. parahaemolyticus* and *L. monocytogenes* could grow under aerobic and anaerobic conditions (Gomez-Gil et al., 2003; Valimaa et al., 2015). However, the modified atmosphere packaging (MAP) also dramatically reduced the growth of Gram-negative bacteria including *Vibrio* spp. (Paludan-Muller et al., 1998). In addition, the Gram-positive *L. monocytogenes* were inhibited under all MAP compositions [(1): 40% CO_2_/55% N_2_/5% O_2_, (2): 60% CO_2_/40% N_2_, and (3): 50% CO_2_/50% N_2_] (Arvanitoyannis et al., 2011). Therefore, the modified atmospheres are an important way of affecting the growth of Gram-negative and Gram-positive bacteria.

The biofilm formation ability of *V. parahaemolyticus* and *L. monocytogenes* in multiple conditions (temperatures, nutrients, contact surface, or pH) has been well studied in previous studies (Di Bonaventura et al., 2008; Nilsson et al., 2011; Bonsaglia et al., 2014; Song et al., 2016). However, few studies paid attention to the stress responses of the biofilm formation of *V. parahaemolyticus* and *L. monocytogenes* to the modified atmospheres. On this basis, the aim of this study was to evaluate the biofilm formation of *V. parahaemolyticus* and *L. monocytogenes* under the stress of different modified atmospheres including anaerobiosis (20% carbon dioxide, 80% nitrogen), micro-aerobiosis (20% oxygen, 80% nitrogen), and aerobiosis (60% oxygen, 40% nitrogen), by determining the changes in the EPS, structural parameters, and the related regulatory genes. The present study will provide a novel insight to unlock the efficient control of Gram-negative and Gram-positive bacteria in modified-atmosphere packaged food.

## Materials and Methods

### Bacterial Strains and Culture Conditions

Four-strain cocktails of *V. parahaemolyticus* strains (VP-S36, VP-39, VP-49, and VP-54) were used (Li et al., 2017). Meanwhile, four-strain cocktails of *L. monocytogenes* strains (NO.12, NO.14, NO.51, and NO.66) were used. All of the genotypes and origins of each strain are listed in Table 1. Strains of *V. parahaemolyticus* were stored at −80°C in tryptic soy broth (TSB, Land Bridge Technology, China) with 50% (v/v) glycerol. And the four *L. monocytogenes* strains in Brain Heart Infusion (BHI) with 50% glycerol were stored at −80°C. Before every experiment, frozen cells of *V. parahaemolyticus* were streaked on thiosulfate citrate bile salts sucrose agar (TCBS agar, Land Bridge Technology, Beijing, China), and *L. monocytogenes* were streaked on PALCAM medium base (Land Bridge Technology, Beijing, China). After that, the working solutions of *V. parahaemolyticus* and *L. monocytogenes* were incubated in TSB (Beijing Land Bridge streaked on Technology Company Ltd., Beijing, China) at 37°C for approximately 12 h and with shaking at 200 r/min. Subsequently, the cultures of *V. parahaemolyticus* and *L. monocytogenes* were diluted by TSB to acquire a bacteria population of 8 log CFU/ml.

**TABLE 1A:** The genotype and origins of *Vibrio parahaemolyticus* used in this study.

**Strains**	**Genotype**	**Origin**
VP-S36	*tdh^–^/trh^–^/tlh^+^*	Shrimp
VP-C39	*tdh^+^/trh^+^/tlh^+^*	CDC
VP-C49	*tdh^+^/trh^+^/tlh^+^*	Clinical isolation
VP-C54	*tdh^+^/trh^+^/tlh^+^*	Clinical isolation

**TABLE 1B:** The genotype and origins of *Listeria monocytogenes* used in this study.

**Strains**	**Genotype**	**Origin**	**Number**
NO.12	1/2a	Raw pork	4bLM
NO.14	1/2a	Human	ATCC 7644
NO.51	4b	Spinal fluid of child with meningitis	ATCC 13932
NO.66	4b	Human	ATCC 19115

### Biofilm Formation in Different Modified Atmosphere Conditions

Biofilm formation of *V. parahaemolyticus* and *L. monocytogenes* cocktails was carried out as described previously (Song et al., 2016; Han et al., 2017) with minor modifications. Static biofilms were grown in 24-well polystyrene microtiter plates (Sangon Biotech Co., Ltd., Shanghai, China). Ten microliters of the obtained *V. parahaemolyticus* and *L. monocytogenes* solutions was added into 990 μl fresh TSB medium, respectively. And then, the OD_600__nm_ values of these two mixtures were determined to be 0.061 and 0.053, respectively. Finally, the bacterial solutions were employed to form biofilms. And each 24-well polystyrene microtiter plate was transferred to sterile sampling bags (32^∗^20 cm) with poor atmosphere permeability (Qingdao Hope Bio-Technology Co., Ltd., Qingdao, China), which were packed by an external vacuum inflatable packaging machine (Qingpa Food Packaging Machinery Co., Ltd., Shanghai, China) under three different atmosphere conditions: anaerobiosis (20% carbon dioxide, 80% nitrogen), micro-aerobiosis (20% oxygen, 80% nitrogen), and aerobiosis (60% oxygen, 40% nitrogen), and then sealed with thermoplastic, respectively. Subsequently, the 24-well plates of *V. parahaemolyticus* cocktail were incubated at 25°C statically to form biofilms for 48 h, and the *L. monocytogenes* cocktail was incubated at 37°C statically to form biofilms for 72 h.

### Crystal Violet Staining Method

Biofilm production was quantified by using the crystal violet staining method as described previously (Han et al., 2017; Tan et al., 2018) with some modifications. After incubation, the supernatant of the plates’ wells was discarded, these plates were gently washed three times with 1 ml of 0.01 M phosphate buffer (PBS, pH 7.4) to remove planktonic cells, and then the microtiter plates were put into the electric blast drying oven for about 20 min and stained with 1 ml of 0.1% (w/v) crystal violet (Sangon Biotech Co., Ltd., Shanghai, China) for 30 min at room temperature. The plates were then gently washed three times with PBS (Sangon Biotech Co., Ltd., Shanghai, China) to remove excess crystal violet. The next step was to dissolve the crystal violet-stained biofilm with 1 ml 95% ethanol for 30 min. Biofilm was solubilized using 1 ml of 95% ethanol (Sinopharm Chemical Reagent Co., Ltd., Shanghai, China) for 30 min. The optical density of each well was measured at a wavelength of 600 nm (Han et al., 2017). All assays were performed in triplicate in three independent experiments. And at least 12 wells were tested in each experiment.

### Visualization of the Biofilms Using Confocal Laser Scanning Microscopy

Before the experiment of cultivating biofilm, a sterile glass slide (1.4 cm in diameter) also needed to be placed into the wells of the 24-well microtiter plates. The culture process of biofilms is as described above. The biofilms of *V. parahaemolyticus* and *L. monocytogenes* cocktails for confocal laser scanning microscopy (CLSM) were pre-treated based on the method of Han et al. (2017). Subsequently, the next step is to add the corresponding bacterial solution and fresh culture medium and then incubate them following the previous experiment of biofilm formation conditions. After static incubation, the biofilms of *V. parahaemolyticus* and *L. monocytogenes* formed on the sterile glass, and then the plates were washed three times with 1 ml of 0.01 M PBS. Next, the plates were fixed with 4% glutaraldehyde (Sangon Biotech Co., Ltd., Shanghai, China) at 4°C for 30 min. Then, the biofilms were gently rinsed three times with 1 ml of 0.01 M PBS. Therefore, after the above operation, SYBR Green I (Sangon Biotech Co., Ltd., Shanghai, China) was used to stain the biofilms at room temperature and dark conditions for 30 min. Before removing the sterile glass from the plate, the plate needs to be washed three times with 1 ml of 0.01 M PBS. It may take about 20 min to air-dry the sterile glass before the end of the experiment. All microscopy images were captured and acquired using the CLMS machine (LSM710, Carl Zeiss AG, Germany). A 40 × objective was used to monitor SYBR Green I fluorescence excited at 488 nm and emitted at 500–550 nm. Then the most representative place was scanned to provide a stack of horizontal planar images with a z-step of 1 μm. In order to compare the change of the biofilms under different modified atmospheres, the CLSM images were performed by using the ISA-2 software (Professor Haluk Beyenal, Montana State University, United States) to determine the structural parameters [such as biovolume, mean thickness, bio-roughness, and textural entropy (TE)] of biofilms (Beyenal et al., 2004b). For each sample, the representative images of nine separate sites on the glass slide were randomly acquired.

### Visualization of the Biofilms Using Scanning Electron Microscopy

The method of culturing bacterial biofilms is similar to that described above. The biofilms of *V. parahaemolyticus* and *L. monocytogenes* cocktails for CLSM were pre-treated based on the method of Han et al. (2017). Next, the plates were incubated to form bacterial biofilms. After static incubation, the biofilms were washed three times with 1 ml of 0.01 M PBS and then were fixed overnight with 2.5% glutaraldehyde at 4°C. Next, the biofilms were gently washed three times with 1 ml of sterile 0.01 M PBS. Then, the biofilms were dehydrated in an ascending acetonitrile series (30, 50, 70, 80, 90, and 100% twice for 10 min each). Samples were then dried and finally coated with a turbomolecular pumped sputter coater (Q150T ES PLUS, Quorum, United Kingdom). Subsequently, extreme-resolution analytical field emission SEM (JEOL JSM-7800F Prime, Japan) was used to observe the biofilms. The images were acquired for three independent replicates.

### EPS Extraction and Chemical Analysis

Extracellular polymeric substance, which consists of bacterial biofilms, was extracted using the sonication method (Liu et al., 2007; Gong et al., 2009; Han et al., 2017). Firstly, the density of the planktonic cells of the suspended cultures was measured by a BioTek microplate reader at OD_595__nm_. Then, the planktonic cells were removed and discarded after washing three times with 1 ml of 0.01 M PBS. Afterward, the mature biofilms were collected and scrapped in 1 ml 0.01 M KCl solution. The cells were sonicated by a sonicator (VCX 500, SONICS, Newtown, CT, United States) for four cycles of 5 s of run and 5 s of pause at a power level of 3.5 Hz. Afterward, the sonicated suspensions were centrifuged at 4°C, 4000 × *g* for 10 min, and the supernatant was then filtered through a 0.22 mm membrane filter (Sangon Biotech Co., Ltd., Shanghai, China). The amounts of protein, carbohydrate, and eDNA in the filtrate were analyzed. The eDNA was quantified by the Quant-iT^TM^ PicoGreen^®^ dsDNA Reagent and Kits (Life Technologies, Shanghai, China) according to the manufacturer’s instructions (Grande et al., 2015). For extracellular protein, it was quantified by the Stable Lowry Protein Assay Kit (Sangon Biotech Co., Ltd., Shanghai, China), and bovine serum albumin (BSA) was used as a protein standard to perform the calibration curve, which contains slightly more modifications compared with the Lowry method. A certain amount of extracellular polysaccharide in the filtrate was quantified by the phenol–sulfuric acid method (Kim and Park, 2013) and expressed as OD_490__nm_/OD_595__nm_. Each experiment was carried out at least three times.

### Expression of Biofilm Formation, EPS, and Virulence-Related Genes

After the treatment of *V. parahaemolyticus* and *L. monocytogenes* biofilm under three modified atmospheric conditions, the total RNA was extracted using the Bacteria RNA Extraction Kit (Vazyme Biotech Co., Ltd., Nanjing, China) and quantified using a BioTek microplate reader. Total RNA was then resuspended in 50 μl of diethyl pyrocarbonate (DEPC)-treated water. RNA purity and integrity were assessed according to a previous study (Kim et al., 2016). Complementary DNA (cDNA) was reversely transcribed from 2 μl of total RNA using a HiScript^®^ III RT SuperMix for qPCR (+ gDNA wiper) (Vazyme Biotech Co., Ltd., Nanjing, China). The qRT-PCR reactions were executed via a ChamQTM Universal SYBR^®^ qPCR Master Mix and carried out by a 7500 Fast Real-Time PCR system (Applied Biosystems, Waltham, MA, United States). All the primers were synthesized by Sangon Biotech (Shanghai, China). All qPCR reactions were performed in a total volume of 20 μl containing 10 μl of 2 × ChamQ Universal SYBR qPCR Master Mix (Vazyme Biotech Co., Ltd., China), 0.4 μl of 10μ M forward primer, 0.4 μl of 10 μM reverse primer, 2 μl of cDNA, and 7.2 μl of deionized water. Cycling parameters for qPCR included an initial denaturation at 95°C for 5 min, followed by 35 cycles of 95°C for 30 s, 58°C for 30 s, and primer extension at 72°C for 30 s. The fluorescent products were detected after the extension step of each cycle. The changes in relative gene expression were calculated with the 2^–ΔΔCT^ method. Primers used in this study for the detection of *V. parahaemolyticus* and *L. monocytogenes* are listed in Table 2. Genes 1–14 in Table 2 belong to *V. parahaemolyticus*, and genes 15–26 belong to *L. monocytogenes*. Each experiment was carried out at least three times.

**TABLE 2:** Primer sequences of the RT-qPCR assay.

**Number**	**Gene**	**Primer name**	**Primer sequence (5′–3′)**	**References**
1.	*recA*	*recA*-F	GCTAGTAGAA AAAGCGGGTG	Ma et al., 2015
		*recA*-R	GCAGGTGCTTC TGGTTGAG	
2.	*oxyR*	*oxyR*-F	TCGTCAGCT AGAGGAAGG	Chung et al., 2016
		*oxyR*-R	TGGTCGCGT AAGCAATGC	
3.	*aphA*	*aphA*-F	ACACCCAACCGT TCGTGATG	Wang et al., 2013
		*aphA*-R	GTTGAAGGCG TTGCGTAGTAAG	
4.	*luxS*	*luxS*-F	GATGGGATGTC GCACTGGTTT	Wang et al., 2014
		*luxS*-R	ACTTGCTGTT CAGAAGGCGTA	
5.	*opaR*	*opaR*-F	TGTCTACCAAC CGCACTAACC	Zhang et al., 2016
		*opaR*-R	GCTCTTTCAAC TCGGCTTCAC	
6.	*mshA*	*mshA*-F	GGTTTCGT TTAGGTCACG	Shime-Hattori et al., 2006
		*mshA*-R	CGTCGAAATG TCGGCGG	
7.	*cpsA*	*cpsA*-F	GCGCACAACGAAG AATATCG	This study
		*cpsA*-R	CCATCTTATC GAGCGTGTCG	
8.	*cpsC*	*cpsC*-F	TCGACCAGGACT GACGATAGAA	This study
		*cpsC*-R	AACCGTGCGC TGCATACTTA	
9.	*cpsR*	*cpsR*-F	CCAATATGCG AACGGACTCA	This study
		*cpsR*-R	AGCACGCCAAG AGACGGTAT	
10.	*vcrD1*	*vcrD1*-F	AAGGTAGGGC AACGCAAAGA	Ding et al., 2016
		*vcrD1*-R	AGCAGCACGAC AGCAATACT	
11.	*vopS*	*vopS*-F	TAGAACGCGATTA CCGTGGG	Ding et al., 2016
		*vopS*-R	TTACCGAGGT CTTTGTCCGC	
12.	*vopD1*	*vopD1*-F	GCGGGTGCAGT AAAAAGCAA	Ding et al., 2016
		*vopD1*-R	AAGCTCACCCA TCAGGTTCG	
13.	*vcrD2*β	*vcrD2*β-F	AGAGAGTTTGGGG ACAAGCG	Ding et al., 2016
		*vcrD2*β-R	CCTTCAGCCG AGCTTTGAGA	
14.	*vopP2*β	*vopP2*β-F	AGAAGGCGGG GTTAAATGCT	Ding et al., 2016
		*vopP2*β-R	ACCTCCGCAACC TAAGTTCA	
15.	*Gap*	*Gap*-F	AAAGCTGGCGCTA AAAAAGTTG	Mattila et al., 2011
		*Gap*-R	TTCATGGTTTACATT GTAAACGATTG	
16.	*flaA*	*flaA*-F	GAAGGCATGACTC AAGCGCA	This study
		*flaA*-R	GCAAGACCAGCAG CGTCATC	
17.	*flgE*	*flgE*-F	CAGCAGGTTCC CCGACTTC	This study
		*flgE*-R	CGGCCTTGTA GTGCTGCAT	
18.	*degU*	*degU*-F	GGAGGAGTAGTCA TTATGGC	This study
		*degU*-R	ACTTCTGGTTG TTGGTAGCC	
19.	*inlA*	*inlA*-F	AAGTGACGTAAGCT CACTTGC	This study
		*inlA*-R	TGTTGGTGGTG TAGGTTCTTG	
20.	*plcA*	*plcA*-F	TTAGCGAGAAC GGGACCAT	This study
		*plcA*-R	CCTTCAGCCG AGCTTTGAGA	
21.	*lmo2504*	*lmo2504*-F	CGCTAAGAGTGC CGCTGTTG	This study
		*lmo2504*-R	TGGCCTCCACCGG AACTTAC	
22	*lmo2554*	*lmo2554*-F	AAAGGCGACGAT GGTTCTGC	This study
		*lmo2554*-R	ACATTTGCGAC ACACAGGGT	
23.	*rmlB*	*rmlB*-F	TATTTCTGTGGAAGC GGGTGT	This study
		*rmlB*-R	CTTTGCGCGTGAT TTTAGTGG	
24.	*actA*	*actA*-F	GGCGAAAGAGT CACTTGC	This study
		*actA*-R	GTTGGAGGCGGT GGAAAT	
25.	*prfA*	*prfA*-F	TAACCAATGGGAT CCACAAG	This study
		*prfA*-R	TGCTAACAGCTG AGCTATGTG	
26.	*hly*	*hly*-F	TGTAAACTTCGGC GCAATC	This study
		*hly*-R	TAAGCAATGGG AACTCCTG	

### Statistical Analysis

The experimental data were expressed as the mean ± standard deviation. Analysis of one-way ANOVA was used to compare the value differences (*P* < 0.05) using SPSS 17.0 (SPSS Inc., Chicago, IL, United States).

## Results

**FIGURE 1:**
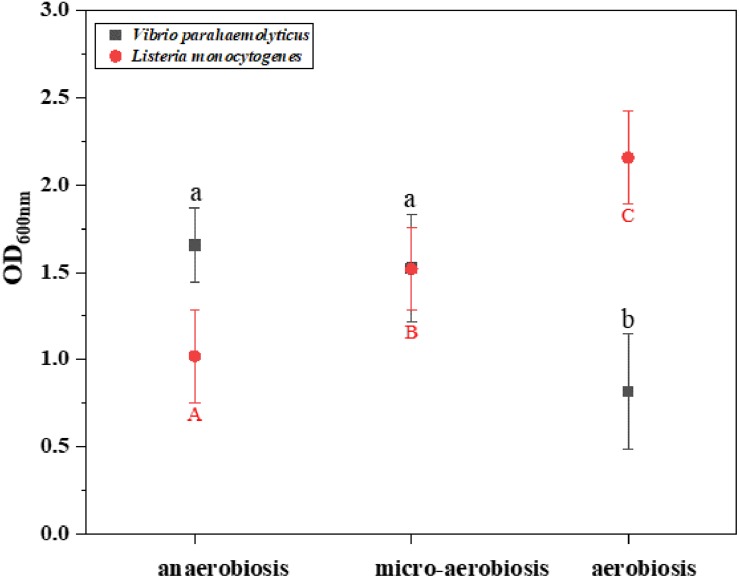
Difference of the biomass (OD_600 nm_) of *Vibrio parahaemolyticus* and *Listeria monocytogenes* by crystal violet staining method under different modified atmospheres: anaerobiosis (20% carbon dioxide, 80% nitrogen), micro-aerobiosis (20% oxygen, 80% nitrogen), and aerobiosis (60% oxygen, 40% nitrogen). The error bar represents the standard deviation of triplicate experiments. Different letters represent statistically significant differences (*P* < 0.05). Black small letters represent *V. parahaemolyticus*, and red capital letters represent *L. monocytogenes*.

The effects of different modified atmospheres against the biofilm formation of *V. parahaemolyticus* and *L. monocytogenes* cocktails are shown in Figure 1. The crystal violet staining assay indicated that the biofilms of *V. parahaemolyticus* were reduced from 1.66 (OD_600__nm_) under anaerobiosis to 1.52 (OD_600__nm_) under micro-aerobiosis, and further significantly (*P* < 0.05) decreased to 0.82 (OD_600__nm_) under aerobiosis. However, the biofilms of *L. monocytogenes* treated by modified atmospheres were significantly (*P* < 0.05) increased from 1.12 (OD_600__nm_) under anaerobiosis to 1.52 (OD_600__nm_) under micro-aerobiosis, and further significantly (*P* < 0.05) increased to 2.15 under aerobiosis. All these facts implied that the modified atmospheres produced completely opposite effects on the biofilm formation of *V. parahaemolyticus* and *L. monocytogenes.*

**FIGURE 2:**
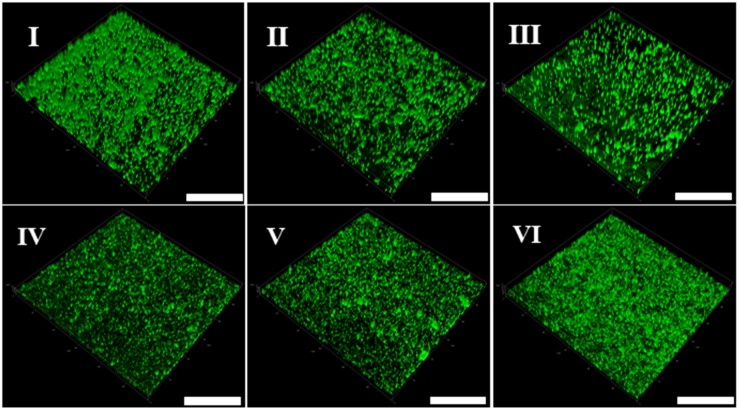
Representative confocal laser scanning microscopy (CLSM) images of biofilm formed by *V. parahaemolyticus* **(I–III)** and *L. monocytogenes* **(IV–VI)** under different modified atmospheres: anaerobiosis (20% carbon dioxide, 80% nitrogen), micro-aerobiosis (20% oxygen, 80% nitrogen), and aerobiosis (60% oxygen, 40% nitrogen). The scale bar represents 100 μm. The images are representative of three independent replicates.

In addition, the CLSM was used to perform *in situ* characterization of *V. parahaemolyticus* and *L. monocytogenes* biofilms under different modified atmospheres. As shown in Figure 2I, the biofilms of *V. parahaemolyticus* presented a compact and structured biofilm architecture under anaerobiosis. However, the biofilms became slightly loose and presented unevenly dispersed structures under micro-aerobiosis (Figure 2II). Much sparser and lower amounts of biofilms were observed under aerobiosis (Figure 2III). For the biofilms of *L. monocytogenes*, they displayed sparser and looser biofilms with a patchy coverage on the contact surface under anaerobiosis (Figure 2IV). Nevertheless, the biofilms presented an increased amount and compact structures under micro-aerobiosis (Figure 2V). Furthermore, the biofilms displayed more compact and dense structures under aerobiosis (Figure 2VI).

**FIGURE 3:**
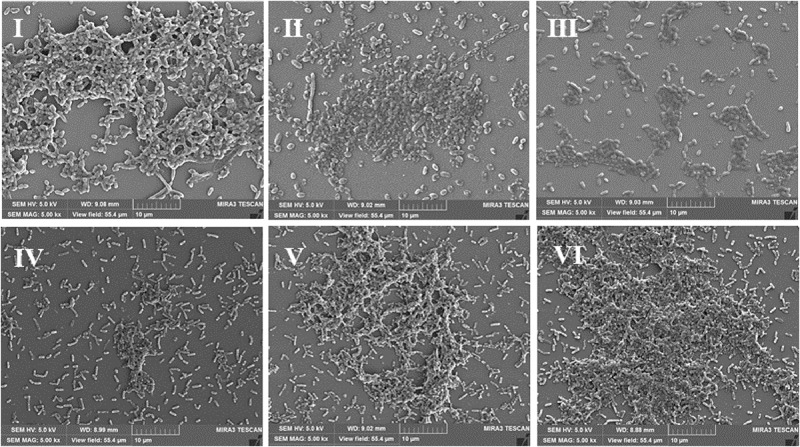
Representative scanning electron microscopy (SEM) images of biofilms formed by *V. parahaemolyticus* **(I–III)** *and L. monocytogenes* **(IV–VI)** under different modified atmospheres: anaerobiosis (20% carbon dioxide, 80% nitrogen), micro-aerobiosis (20% oxygen, 80% nitrogen), and aerobiosis (60% oxygen, 40% nitrogen). The scale bar represents 10 μm. The images are representative of three independent replicates.

Scanning electron microscopy (SEM) is used to directly observe biofilms (Pan et al., 2016). As shown in Figure 3I, a layer of mature biofilms of *V. parahaemolyticus* with well-organized network structures was observed under anaerobic conditions, and bacterial biofilm cells were closer to each other. However, few bacterial cells aggregated, and the ordered biofilm structures were not found under micro-aerobiosis (Figure 3II). Even much fewer cells were sporadically scattered on the contact surface under aerobiosis (Figure 3III). The biofilms of *L. monocytogenes* also exhibited an opposite trend of structure changes compared with *V. parahaemolyticus* (Figures 3IV–VI). Based on above results, the modified atmospheres especially for aerobiosis significantly inhibited the biofilm formation of *V. parahaemolyticus*, while the anaerobiosis greatly restrained the biofilm formation of *L. monocytogenes*.

**FIGURE 4:**
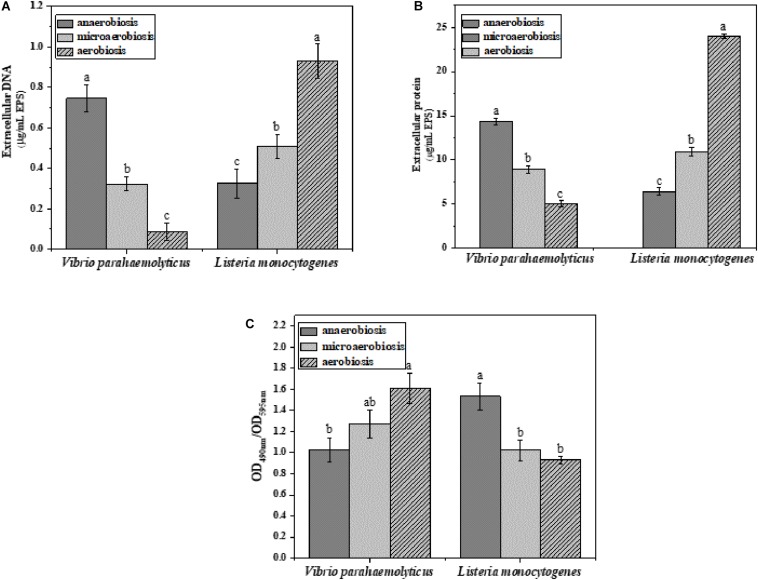
Chemical composition and contents of EPS in *V. parahaemolyticus and L. monocytogenes* biofilms under different modified atmospheres: anaerobiosis (20% carbon dioxide, 80% nitrogen), micro-aerobiosis (20% oxygen, 80% nitrogen), and aerobiosis (60% oxygen, 40% nitrogen). **(A)** Extracellular DNA (μg/ml), **(B)** extracellular protein (μg/ml), and **(C)** polysaccharide (OD_490__nm_/OD_595__nm_) in EPS of *V. parahaemolyticus* and *L. monocytogenes* biofilms. Error bars represent the standard error. And the same letter represents no significant difference (*P* < 0.05).

The effects of modified atmospheres on the production of EPS including eDNA, protein, and polysaccharide were examined in the biofilms of *V. parahaemolyticus* and *L. monocytogenes* (Figure 4). Overall, the eDNA and protein of *V. parahaemolyticus* biofilms were greatly reduced from anaerobic to aerobic conditions. However, the extracellular polysaccharide was increased. In detail, the eDNA of EPS was significantly (*P* < 0.05) decreased from 0.75 μg/ml under anaerobiosis to 0.08 μg/ml (89%) under aerobiosis (Figure 4A). Meanwhile, the extracellular protein of EPS was markedly (*P* < 0.05) reduced from 14.3 μg/ml under anaerobiosis to 5.0 μg/ml (65%) under aerobiosis (Figure 4B). Conversely, the extracellular polysaccharide of EPS in *V. parahaemolyticus* biofilms was slightly increased from 1.02 to 1.60 (OD_490__nm_/OD_595__nm_) (Figure 4C).

For the biofilms of *L. monocytogenes*, the eDNA and protein were dramatically elevated from anaerobic to aerobic conditions. However, the extracellular polysaccharide was reduced from anaerobic to aerobic conditions. In Figure 4A, the eDNA content of EPS was significantly (*P* < 0.05) increased from 0.32 μg/ml under anaerobiosis to 0.93 μg/ml (73%) under aerobiosis. Similarly, the extracellular protein of EPS was markedly (*P* < 0.05) increased from 6.42 μg/ml under anaerobiosis to 24 μg/ml (66%) under aerobiosis. Conversely, the extracellular polysaccharide of EPS in *L. monocytogenes* biofilms was gradually reduced from 1.53 under anaerobiosis to 0.93 under aerobiosis (OD_490__nm_/OD_595__nm_). It was concluded that the modified atmosphere treatment inhibited the biofilm formation of *V. parahaemolyticus*, and the inhibition degree values could be ranked as eDNA > extracellular proteins > extracellular polysaccharide; however, the modified atmosphere treatment enhanced the biofilm formation of *L. monocytogenes*, and the enhancement degree values could be ranked as extracellular proteins > eDNA > extracellular polysaccharide.

**FIGURE 5:**
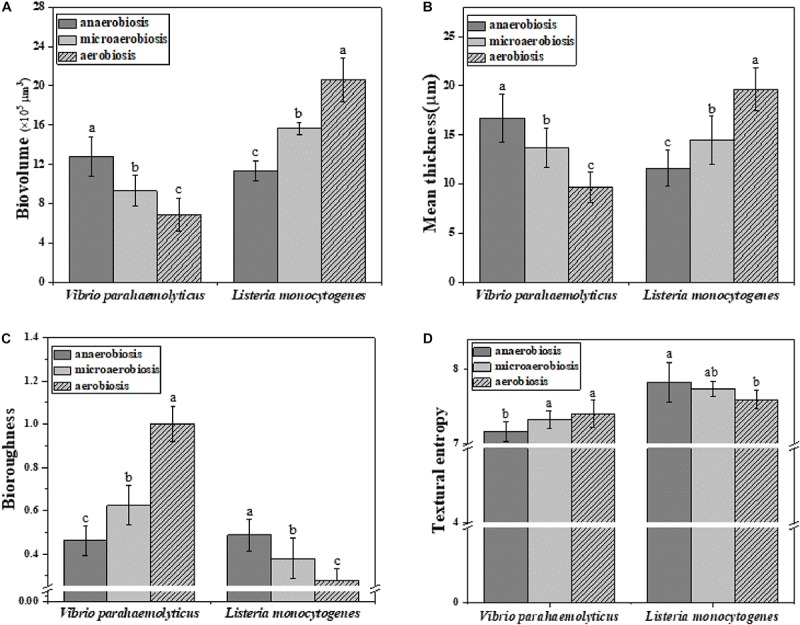
Quantification of structural parameters in biofilm formed by *V. parahaemolyticus* and *L. monocytogenes* biofilms under different modified atmospheres: anaerobiosis (20% carbon dioxide, 80% nitrogen), micro-aerobiosis (20% oxygen, 80% nitrogen), and aerobiosis (60% oxygen, 40% nitrogen). **(A)** Biovolume, **(B)** Mean thickness, **(C)** Bio-roughness, and **(D)** Textural entropy. Error bars represent the standard error. And the same letter represents no significant difference (*P* < 0.05).

Subsequently, the morphological and structural changes of the biofilms of *V. parahaemolyticus* and *L. monocytogenes* were analyzed by ISA software (Figure 5). The quantitative image analysis (QIA) of *V. parahaemolyticus* biofilms revealed that the biovolume of biofilms was greatly (*P* < 0.05) decreased from 13.49 × 10^5^ μm^3^ under anaerobiosis to 8.62 × 10^5^ μm^3^ under aerobiosis (Figure 5A). The biofilm thickness was significantly (*P* < 0.05) reduced from 16.72 μm under anaerobiosis to 9.65 μm under aerobiosis (Figure 5B). Meanwhile, the bio-roughness was significantly (*P* < 0.05) increased from 0.46 under anaerobiosis to 1.00 under aerobiosis (Figure 5C). Meanwhile, the TE was slowly increased from 7.16 under anaerobiosis to 7.40 under aerobiosis (Figure 5D).

For *L. monocytogenes*, the biovolume of its biofilms was significantly (*P* < 0.05) increased from 11.41 × 10^5^ to 20.58 × 10^5^ μm^3^ during the physiological conversion from anaerobiosis to aerobiosis (Figure 5A). The biofilm thickness was dramatically (*P* < 0.05) increased from 11.63 to 19.63 μm (Figure 5B). Meanwhile, its bio-roughness was markedly (*P* < 0.05) improved from 0.49 under anaerobiosis to 0.27 under aerobiosis (Figure 5C). The TE was gradually decreased from 7.82 under anaerobiosis to 7.59 under aerobiosis (Figure 5D).

**FIGURE 6:**
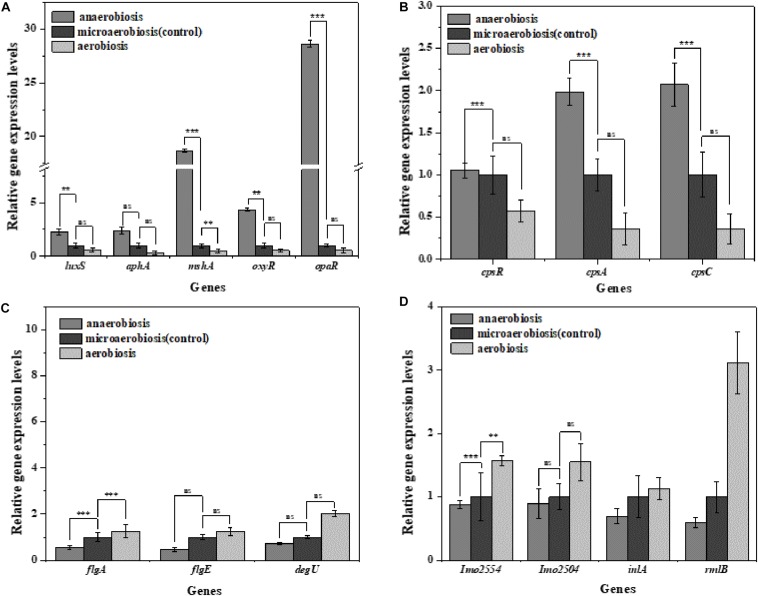
Fold change normalized to reference gene *recA* [*V. parahaemolyticus* **(A,B)**] and *Gap* [*L. monocytogenes* **(C,D)**] in the expression of biofilm and extracellular polymeric substance (EPS) genes in: anaerobiosis (20% carbon dioxide, 80% nitrogen) and aerobiosis (60% oxygen, 40% nitrogen) relative to micro-aerobiosis (20% oxygen, 80% nitrogen). Error bars represent the standard error. Asterisks represent statistically significant differences (*P* < 0.05) in fold change relative to micro-aerobiosis.

*Vibrio parahaemolyticus* possesses the ability to form biofilms and secrete endotoxin, which can lead to serious diseases, and *L. monocytogenes* can also form biofilms and cause terrible listeriosis by secreting exotoxin such as listeriolysin O (LLO) (Gekara et al., 2008; Hamon et al., 2012; Wang et al., 2015). In this study, the regulatory genes of biofilm formation (*luxS*, *aphA*, *mshA*, *oxyR*, and *opaR*) and EPS production genes (*cpsA*, *cpsC*, and *cpsR*) of *V. parahaemolyticus* were selected to determine the effects of the modified atmospheres on their transcriptional levels. In Figure 6A, the expression levels of the biofilm formation genes and EPS production genes were slightly downregulated by aerobiosis; however, all the genes were significantly (*P* < 0.05) upregulated under anaerobiosis. For the *L. monocytogenes* cells, the gene expression levels of the biofilm formation (*flgA*, *flgE*, and *degU*) and EPS production (*Imo2554*, *Imo2504*, *inlA*, *rmlB*) were significantly (*P* < 0.05) downregulated under the anaerobiosis, except *inlA* and *rmlB* (Figure 6C). However, all the above genes were slightly upregulated by aerobiosis, except the obviously upregulated *flgA*.

**FIGURE 7:**
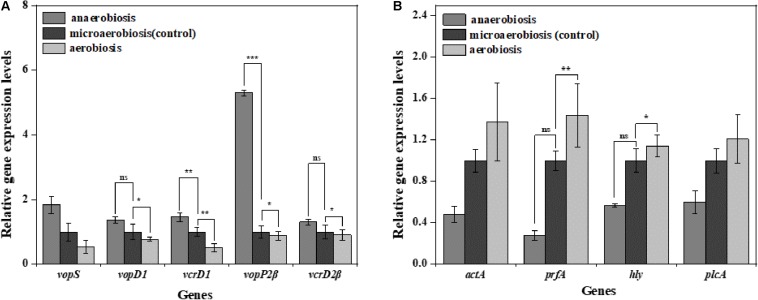
Fold change normalized to reference gene *recA* [*V. parahaemolyticus* **(A)**] and *Gap* [*L. monocytogenes* **(B)**] in the expression of virulence genes in: anaerobiosis (20% carbon dioxide, 80% nitrogen) and aerobiosis (60% oxygen, 40% nitrogen) relative to micro-aerobiosis (20% oxygen, 80% nitrogen). Error bars represent the standard error. Asterisks represent statistically significant differences (*P* < 0.05) in fold change relative to micro-aerobiosis.

To investigate the effects of modified atmospheres on the expression levels of virulence genes, the genes including *vopS*, *vopD1*, *vcrD1*, *vopP2*β, and *vcrD2*β of *V. parahaemolyticus* and *actA*, *plfA*, *hly*, and *plcA* of *L. monocytogenes* were evaluated by RT-qPCR. For *V. parahaemolyticus* (Figure 7A), the expression levels of all the virulence genes were downregulated under aerobiosis, reaching 1.88, 1.30, 1.96, 1.14, and 1.10 times, separately; but all the genes were upregulated under anaerobiosis, reaching 1.84, 1.36, 1.46, 5.30, and 1.31 times, respectively. However, the expression levels of all virulence genes of *L. monocytogenes* were downregulated under anaerobiosis, reaching 2.09, 3.58, 1.76, and 1.66 times, respectively; however, all the genes were upregulated under aerobiosis, reaching 1.37, 1.44, 1.14,and 1.21 times, separately (Figure 7B).

## Discussion

Previous studies showed that all the tested modified atmospheres were effective in reducing *V. parahaemolyticus* in sea bream fillets compared with air-packaged samples (Provincial et al., 2013a). Meanwhile, Provincial et al. (2013b) found that the modified atmospheres were more effective in decreasing the *Listeria* spp. with the increase of CO_2_ concentrations from 20% to 30%. Rutherford et al. (2007) also found differences in the amount of *Listeria* on shrimp which were stored in air, vacuum, and map packaging (100% CO_2_) at 3, 7, and 10°C. Moreover, *V. parahaemolyticus* and *L. monocytogenes* could form bacterial biofilms to survive on food processing surfaces under anaerobiosis or aerobiosis (Voidarou et al., 2011; Valimaa et al., 2015; Perez-Ibarreche et al., 2016; Song et al., 2016). In an early study, the biofilm formation of *Vibrio vulnificus* was reduced due to the lack of oxygen (Phippen and Oliver, 2015). However, *Salmonella* spp. produced the highest amount of biofilm under a CO_2_-rich atmosphere (Stepanovic et al., 2003). In food products, Wang et al. (2017) reported that the MAP inhibited the growth of *Pseudomonas fragi* strains in meat, and more loose and less bound EPS were produced by *P. fragi* in the modified-atmosphere packaged samples. The present study demonstrated that *V. parahaemolyticus* possessed a stronger ability to develop biofilms under anaerobic conditions in comparison with micro-aerobiosis and aerobiosis (Figure 1).

For Gram-positive bacteria, compared to the increased CO_2_ atmosphere and anaerobic condition, the aerobic atmosphere of methicillin-resistant *Staphylococcus aureus* was not efficient in promoting the biomass of biofilms (Ursic and Tomic, 2008). However, the aerobic biofilms of wild-type *S. aureus* were more robust than that under anaerobic conditions (Hess et al., 2013). Our results showed that the *L. monocytogenes* biofilms were increased from 1.01 (OD_600__nm_) under anaerobiosis to 2.02 (OD_600__nm_) under aerobiosis (Figure 1). In addition, the biofilms formed by different Gram-negative *Salmonella* strains (*n* = 30) exhibited a different stress response to the modified atmospheres, indicating the heterogeneity of the biofilm formation ability of *Salmonella* strains under the modified atmospheres (Stepanovic et al., 2003).

It is widely reported that high fluorescence intensities observed by CLSM are caused by the aggregation of biofilms in different layers and depths (Webster et al., 2005; Han et al., 2017). Moreover, SEM images can also be used to describe the biofilm morphotypes (Simões et al., 2007). In our study, the results of CLSM and SEM clearly showed that the biofilm cells of *V. parahaemolyticus* displayed more compact aggregates and well-organized structures under anaerobiosis compared to micro-aerobiosis and aerobiosis (Figures 2, 3). However, it was observed that few aggregated cells and sparser structures of *L. monocytogenes* biofilms formed with a lower fluorescence intensity under anaerobiosis (Figures 2, 3). Overall, the results of the CLSM and SEM confirmed the results of crystal violet staining.

The EPS consists of about 80% dry mass of the biofilms, mainly including extracellular proteins (3–37%), nucleic acids (9–50%), and polysaccharides (3–21%) (Andersson et al., 2009), which plays a major role in mediating biofilm formation (Liu et al., 2007). It was concluded that the eDNA was essential in the initial establishment of *Pseudomonas aeruginosa* biofilms and perhaps biofilms formed by other bacteria (Whitchurch et al., 2002). Besides, three matrix proteins contributing to biofilm stability were identified in *Vibrio cholerae*, which involved in cell–cell and cell–surface adhesion (Yildiz et al., 2014). Up to now, the most descriptive matrix protein was extracellular adhesin CdrA, which promoted the aggregate formation through the interaction of extracellular polysaccharide Psl under planktonic conditions and helped to stabilize the matrix and maintain the structural integrity of aggregates (da Silva et al., 2019). In Figure 4, the eDNA and extracellular proteins of the *V. parahaemolyticus* biofilms were significantly (*P* < 0.05) reduced from anaerobiosis to aerobiosis, but the extracellular polysaccharides were gradually increased. However, our results showed that the eDNA and extracellular proteins of the *L. monocytogenes* biofilms were dramatically (*P* < 0.05) increased from anaerobiosis to aerobiosis; conversely, the extracellular polysaccharides were decreased (Figure 4). Our results were supported by the fact that anaerobic incubation decreased the production of extracellular polysaccharide in Gram-negative *Escherichia coli* 0157:H7 (Dewanti and Wong, 1995). It showed that the extracellular proteins and eDNA of Gram-positive *S. aureus* were significantly decreased under anaerobic conditions (Hess et al., 2013). The results demonstrated that the anaerobic conditions induced the highest levels of carbohydrates in biofilms compared to aerobic conditions (Yang et al., 2006). The reported results were highly coincident with the changes of EPS of Gram-positive *L. monocytogenes* in this study.

Quantitative image analysis was used to characterize the structure of the biofilms (Tan et al., 2018). The variation of the textural parameters reflects the changes of biofilm structure and biofilm adhesion ability (Figures 5A–C). The biovolume represented the total volume of cells (μm^3^) in the biofilms (Bridier et al., 2010). Meanwhile, the mean thickness (μm) of biofilms was also determined directly from the confocal stack images. In Figures 5A,B, the decrease in biovolume and thickness of biofilms suggested that the total volume of adherent cells was reduced from anaerobiosis to aerobiosis. Bio-roughness offered a metric of variations in biofilm thickness and was an indicator of the superficial biofilm-interface heterogeneity, so the significantly increased bio-roughness suggested that a high variation of biofilm thickness means a high superficial biofilm-interface heterogeneity (Bridier et al., 2010) (Figure 5C). It is now widely accepted that the texture entropy (TE) is positively correlated with the heterogeneity of biofilms (Beyenal et al., 2004a), but the changes of TE of these two bacterial biofilms were not obvious, indicating that the heterogeneity of biofilms did not exhibit obvious alteration under modified atmosphere treatment (Figure 5D). However, the increase in biovolume and thickness of *L. monocytogenes* biofilms indicated the increased total volume of adherent cells from anaerobiosis to aerobiosis. The bio-roughness, an indicator of variation of biofilm thickness, was significantly (*P* < 0.05) decreased, implying the low superficial heterogeneity of biofilm interface from anaerobiosis to aerobiosis (Bridier et al., 2010). It was concluded that the modified atmospheres could highly shape the biofilm formation of *V. parahaemolyticus* and *L. monocytogenes* cocktails. Based on the above analysis, this study was the first one to evaluate the potential effects of the modified atmospheres on bacterial biofilms by monitoring the changes of biofilm architectures, chemical compositions, etc.

The biofilm formation of *V. parahaemolyticus* and *L. monocytogenes* was regulated by a variety of genes. For *V. parahaemolyticus*, the *oxyR* has been known to regulate the formation of cell appendages and biofilm formation (Chung et al., 2016). The expression of quorum-controlled genes *aphA* was necessary for the biofilm formation of *V. parahaemolyticus* (Wang et al., 2013). Meanwhile, it has been shown that quorum-controlled genes *opaR* directly or indirectly regulated 5.2% of genes in the genome of *V. parahaemolyticus* which were pertinent to social activities of bacteria such as biofilm formation (Gode-Potratz and McCarter, 2011). Additionally, biofilm formation was also regulated by the *luxS*-dependent quorum-sensing system in *V. parahaemolyticus* (Guo et al., 2018). From anaerobiosis to aerobiosis, our study showed that the expression levels of *oxyR*, *aphA*, *opaR*, and *luxS* were downregulated. Such facts indicated that the bacteria decreased their physiological activity and mitigated the expression of essential genes to cut down the energy costs of cells to adapt the changes of environmental conditions (Bayramoglu et al., 2017). Therefore, the results suggested a relationship between the biofilm formation and expression of biofilm and quorum-sensing genes in *V. parahaemolyticus* (Lamas et al., 2016).

*MshA* is a type IV pilin subunit gene that mediates the adhesion of *V. parahaemolyticus* to the surface through pili (Shime-Hattori et al., 2006; Aagesen and Hase, 2012). The expression of *mshA* significantly increased (*P* < 0.05) under anaerobic conditions but obviously decreased under aerobic conditions, which might suggest decreased functions of type IV pili in the adhesion of *V. parahaemolyticu*s. Meanwhile, the *cpsA* and *cpsC* regulated the production and transportation of CPS, and *cpsR* was required for the exopolysaccharide production of *V. parahaemolyticus* biofilm (Guvener and McCarter, 2003; Zhang et al., 2018). The downregulation of these genes (*cpsA*, *cpsC*, and *cpsR*) suggested that the aerobic conditions inhibited the production of CPS and exopolysaccharide, which would reduce the adhesion ability and biofilm formation of *V. parahaemolyticus* on the target surfaces. Likewise, the virulence genes of *V. parahaemolyticus* are also affected by the modified atmospheres. *VcrD1, vopS*, and *vopD1* are the main virulence factors of the *V. parahaemolyticus* type III secretory system 1 (T3SS1). VcrD1, an inner membrane protein, is a component of T3SS1 in *V. parahaemolyticus* (Kwon-Sam et al., 2004; Noh et al., 2015). *VopS* is an effector secreted by T3SS1 during infection and can inhibit actin assembly (Yarbrough et al., 2009). *VopD1* is the essential component of the translocation of T3SS1 (Shimohata et al., 2012). *VopP2*β and *vcrD2*β are the main virulence factors of *V. parahaemolyticus* type III secretory system 2 (T3SS2). *VopP2*β inhibits mitogen-activated protein kinases (MAPK) signal transduction and prevents ATP binding in T3SS2 (Trosky et al., 2007; Tsai et al., 2013). And *vcrD2*β encodes an inner membrane protein of T3SS2 (Kwon-Sam et al., 2004; Tsai et al., 2013). The type III secretion system (T3SS), which consists of T3SS1 (*vcrD1*, *vopS*, *vopD1*) and T3SS2 (*vopP2*β, *vcrD2*β), is considered to be an important virulence factor for delivering effectors into host cells (Noh et al., 2015). The downregulation of these genes (*VcrD1*, *vopS*, *vopD1 VopP2*β, *vcrD2*β) suggested that the aerobic conditions inhibited the expression of these virulence genes, but the anaerobic conditions conversely improved the expression of virulence genes.

For *L. monocytogenes*, *flgA* and *flgE* are flagellum-related genes associated with biofilm formation (Li R. et al., 2018). The *degU* gene-encoded DegU is essential for flagellar synthesis and bacterial motility in *L. monocytogenes* (Gueriri et al., 2008; Pieta et al., 2014). It is well known that flagellum-mediated motility plays a predominant role in biofilm formation of *L. monocytogenes* (Renier et al., 2011). The upregulation of *flgA*, *flgE*, and *degU* (*P* < 0.05) implied that the aerobic conditions facilitated the initial biofilm formation of *L. monocytogenes*. However, the downregulation of *flgA*, *flgE*, and *degU* indicated that anaerobic conditions caused a decrease in the ability of biofilm formation. L-rhamnose is widely found in cell walls and capsules of many pathogenic bacteria including *L. monocytogene*s, which can be regulated by *rmlB* (Giraud and Naismith, 2000; Eugster et al., 2015). *Imo2554* is confirmed to be involved in the glycolipid synthesis of *L. monocytogenes*, an important cell wall polymer in Gram-positive bacteria (Webb et al., 2009). *Imo2504* encodes the binding protein of the cell wall which participates in the biofilm formation (Lourenco et al., 2013). *InlA* encodes InlA (the bacterial surface protein), which gains invasiveness and invades the *L. monocytogenes* cells (Pizarro-Cerda et al., 2012). The anaerobic downregulation of *rmlb*, *lmo2554*, *lmo2504*, and *inlA* indicated that the synthesis of these key proteins was inhibited, indicating the highly decreased physiological activity of *L. monocytogenes*. Conversely, the aerobic upregulation of these genes suggested the enhanced expression of key proteins and physiological activity of *L. monocytogenes* which finally strengthened the biofilm formation. For the virulence genes, it was concluded that the pathogenesis was mainly related to the expression of virulence genes (LIPI-1) (Stelling et al., 2010). The virulence genes were located in the virulence island, which is a 9-KB-long gene cluster mainly containing *hly*, *plcA*, *prfA*, and *actA* (Stelling et al., 2010). In this study, the anaerobic downregulation of *hly*, *plcA*, *prfA*, and *actA* suggested that the toxicity of *L. monocytogenes* was decreased, while the aerobic upregulation of these genes indicated the increased toxicity of *L. monocytogenes*.

## Conclusion

The modified atmospheres significantly reduced the eDNA and proteins of EPS and negatively altered the biofilm structures of *V. parahaemolyticus* during the physiological conversion from anaerobiosis to aerobiosis. The modified atmospheres also downregulated the expression of biofilm formation genes (*luxS*, *aphA*, *mshA*, *oxyR*, and *opaR*) and EPS production genes (*cpsA*, *cpsC*, and *cpsR*) and virulence genes (*vopS*, *vopD1*, *vcrD1*, *vopP2*β, and *vcrD2*β) of *V. parahaemolyticus*. Conversely, the expressions of biofilm formation genes (*flgA*, *flgU*, and *degU*), EPS production genes (*Imo2554*, *Imo2504*, *inlA*, *rmlB*) and virulence genes (*vopS*, *vopD1*, *vcrD1*, *vopP2*β, and *vcrD2*β) of *L. monocytogenes* were upregulated during the same physiological conversion. Therefore, the modified atmospheres showed significantly different regulation on the biofilm formation of Gram-negative *V. parahaemolyticus* and Gram-positive *L. monocytogenes*. The generated knowledge will facilitate our understanding of the stress response of Gram-negative and Gram-positive pathogens to the modified atmosphere treatment and hence provide an efficient way to control the pathogens in modified-atmosphere packaged food.

## Data Availability Statement

All datasets generated for this study are included in the article.

## Author Contributions

YZ, JW, YP, and HL conceived and supervised the study. HQ and WL designed and performed the experiments. HQ analyzed the data. LG, LT, JW, and YZ revised the manuscript. HQ wrote the manuscript.

## Conflict of Interest

The authors declare that the research was conducted in the absence of any commercial or financial relationships that could be construed as a potential conflict of interest.
